# Acute Three‐Dimensional Hypoxia Regulates Angiogenesis

**DOI:** 10.1002/adhm.202403860

**Published:** 2024-12-02

**Authors:** Dimitris Ntekoumes, Jiyeon Song, Haohao Liu, Connor Amelung, Ya Guan, Sharon Gerecht

**Affiliations:** ^1^ Department of Biomedical Engineering Duke University Durham NC 27708 USA; ^2^ Department of Chemical and Biomolecular Engineering Johns Hopkins University Baltimore MD 21218 USA

**Keywords:** angiogenesis, endothelial cells, hydrogel, hypoxia, reactive oxygen species

## Abstract

Hypoxia elicits a multitude of tissue responses depending on the severity and duration of the exposure. While chronic hypoxia is shown to impact development, regeneration, and cancer, the understanding of the threats of acute (i.e., short‐term) hypoxia is limited mainly due to its transient nature. Here, a novel gelatin‐dextran (Gel‐Dex) hydrogel is established that decouples hydrogel formation and oxygen consumption and thus facilitates 3D sprouting from endothelial spheroids and, subsequently, induces hypoxia “on‐demand.” The Gel‐Dex platform rapidly achieves acute moderate hypoxic conditions without compromising its mechanical properties. Acute exposure to hypoxia leads to increased endothelial cell migration and proliferation, promoting the total length and number of vascular sprouts. This work finds that the enhanced angiogenic response is mediated by reactive oxygen species, independently of hypoxia‐inducible factors. Reactive oxygen species‐dependent matrix metalloproteinases activity mediated angiogenic sprouting is observed following acute hypoxia. Overall, the Gel‐Dex hydrogel offers a novel platform to study how “on‐demand” acute moderate hypoxia impacts angiogenesis, with broad applicability to the development of novel sensing technologies.

## Graphical abstract

In this work, the authors develop a hydrogel platform that rapidly induces acute moderate hypoxia “on‐demand” without compromising its mechanical properties. This platform initially facilitates the establishment of 3D endothelial sprouts that are subsequently exposed to acute hypoxia. The elevated production of reactive oxygen species allows for matrix degradation, enhancing cell migration and proliferation, and promoting angiogenesis from the existing 3D sprouts.

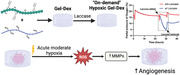

## 1 Introduction

Molecular oxygen (O_2_) is central to myriad biochemical reactions that regulate cellular bioenergetics and sustain life on Earth. Accordingly, the lack of oxygen, or hypoxia (O_2_<5%), is a well‐recognized biochemical signal that plays a key role both in physiological and pathophysiological states.^[^ ^1^, ^2^ ^]^ Depending on its duration, hypoxia is classified as acute, lasting from minutes to a few hours, or chronic, lasting from hours to days or weeks.^[^ ^3^ ^]^ Chronic hypoxia is common in diseases like cancer^[^ ^4^, ^5^, ^6^ ^]^ and cardiovascular disorders.^[^ ^7^, ^8^, ^9^ ^]^ In certain pathologies such as ischemic stroke, localized cessation of blood supply, most commonly caused by a thrombus, can lead to acute hypoxia.^[^ ^10^ ^]^ This ischemia‐driven hypoxia attracts inflammatory cells, further exacerbating tissue damage.^[^ ^11^, ^12^ ^]^ Acute hypoxia poses a threat in stressed environments, even without an underlying condition. In high‐altitude flights and operations, equipment malfunction commonly leads to acute hypoxia due to reduced barometric pressure.^[^ ^13^ ^]^ On such occasions, symptoms of acute hypoxia involve impairment of cognitive function^[^ ^13^, ^14^, ^15^, ^16^ ^]^ which eventually leads to loss of consciousness with increasing altitude. On the other hand, exposure to hypoxia for a few hours and subsequent re‐oxygenation has been shown to have a protective role in enhancing blood vessel density and blood flow after myocardial infarction^[^ ^17^, ^18^, ^19^, ^20^ ^]^ and brain ischemia.^[^ ^21^, ^22^, ^23^, ^24^ ^]^ Nonetheless, the lack of appropriate models to recapitulate acute hypoxia has hindered our understanding of its long‐term impact and the underlying mechanism.

Lining the inner blood vessels, endothelial cells (ECs) are the first to encounter changes in O_2_ concentrations, including rapid drop leading to acute hypoxia. The role of endothelium has only recently emerged as an active regulator of the acute hypoxic response.^[^ ^25^ ^]^ Importantly, in adult tissues, acute hypoxia impacts the already‐established endothelial sprouts rather than individual cells.^[^ ^17^, ^19^, ^24^ ^]^ Thus, elucidating the mechanisms by which human endothelium responds to the rapid drop in O_2_ would identify targets for countermeasures when exposure happens and benefit the development of new sensing technologies that would alert individuals in a timely manner.

Approaches for studying cellular behavior in an O_2_‐modulated environment largely involve the use of hypoxia chambers that are flushed with mixed gasses to achieve the desired O_2_ concentration.^[^ ^26^ ^]^ Despite the user‐defined level of O_2_ (i.e., 1% O_2_), the large volume of the conventional platforms leads to heterogeneity of the gas within the chamber.^[^ ^27^, ^28^ ^]^ Investigating acute hypoxia in vitro is mostly conducted under the assumption that the O_2_ content of the cell medium and the chamber are similar. However, due to low solubility and diffusion limitations, O_2_ levels require a significant amount of time to reach equilibrium,^[^ ^29^ ^]^ offering limited capabilities to temporally control the O_2_ tension within the chamber. Cell‐dependent factors, including proliferation and confluency, significantly influence the pericellular O_2_ concentrations,^[^ ^27^, ^28^ ^]^ making it difficult to predict the precise O_2_ levels experienced by cells based on user‐defined O_2_ settings. In our previous work, we developed O_2_‐controllable hydrogels in which laccase‐mediated dimerization consumes O_2_, allowing hypoxic cell culture in standard incubators, without the need for additional equipment.^[^ ^30^, ^31^, ^32^, ^33^, ^34^ ^]^ These hydrogels offer a 3D microenvironment that enabled us to investigate the effect of chronic hypoxia in guiding vascular morphogenesis^[^ ^30^ ^]^ and cluster‐based vasculogenesis^[^ ^32^, ^33^ ^]^ from single cell suspension of endothelial colony forming cells (ECFCs). In the original approach, we exposed individual ECFCs to low oxygen levels while forming the 3D matrix through oxygen‐consuming polymerization. This setup provided an attractive platform to study how vasculogenesis commences in a hypoxic microenvironment. Nevertheless, to solely assess the effect of hypoxia on promoting angiogenesis, we needed a suitable, non‐hypoxic microenvironment for the initial growth of 3D endothelial sprouts. Therefore, our initial approach was not suitable for studying how vascular sprouts would respond to low O_2_ tensions. In our refined approach, we sought to advance this technology by decoupling hydrogel polymerization and hypoxia induction, allowing us to study how acute hypoxia individually impacts the established endothelial sprouts.

Building on our previous knowledge, we developed a gelatin‐dextran hydrogel system (Gel‐Dex) that induces hypoxia “on‐demand.” Our platform facilitates sprouting from endothelial spheroids and, subsequently, rapidly exposes the established sprouts to moderate hypoxia (≈5–10% O_2_) acutely (3 h) without compromising hydrogel mechanics. We show that acute hypoxic exposure alone enhances cell migration and proliferation, resulting in enhanced angiogenesis from the existing endothelial sprouts. Inhibition studies reveal that the pro‐angiogenic response is reactive oxygen species (ROS), but not HIF‐dependent, possibly due to the duration and severity of the induced hypoxia. We further confirmed the role of matrix metalloproteinases (MMPs) in mediating the acute angiogenic response, using a broad small‐molecule MMP inhibitor. The work developed here sets the foundation for delineating the mechanism by which established vascular sprouts respond to acutely induced hypoxia.

## 2 Results and Discussion

### 2.1 Design and Fabrication of “On‐Demand” Hypoxic Gel‐Dex Hydrogels

**Figure 1:**
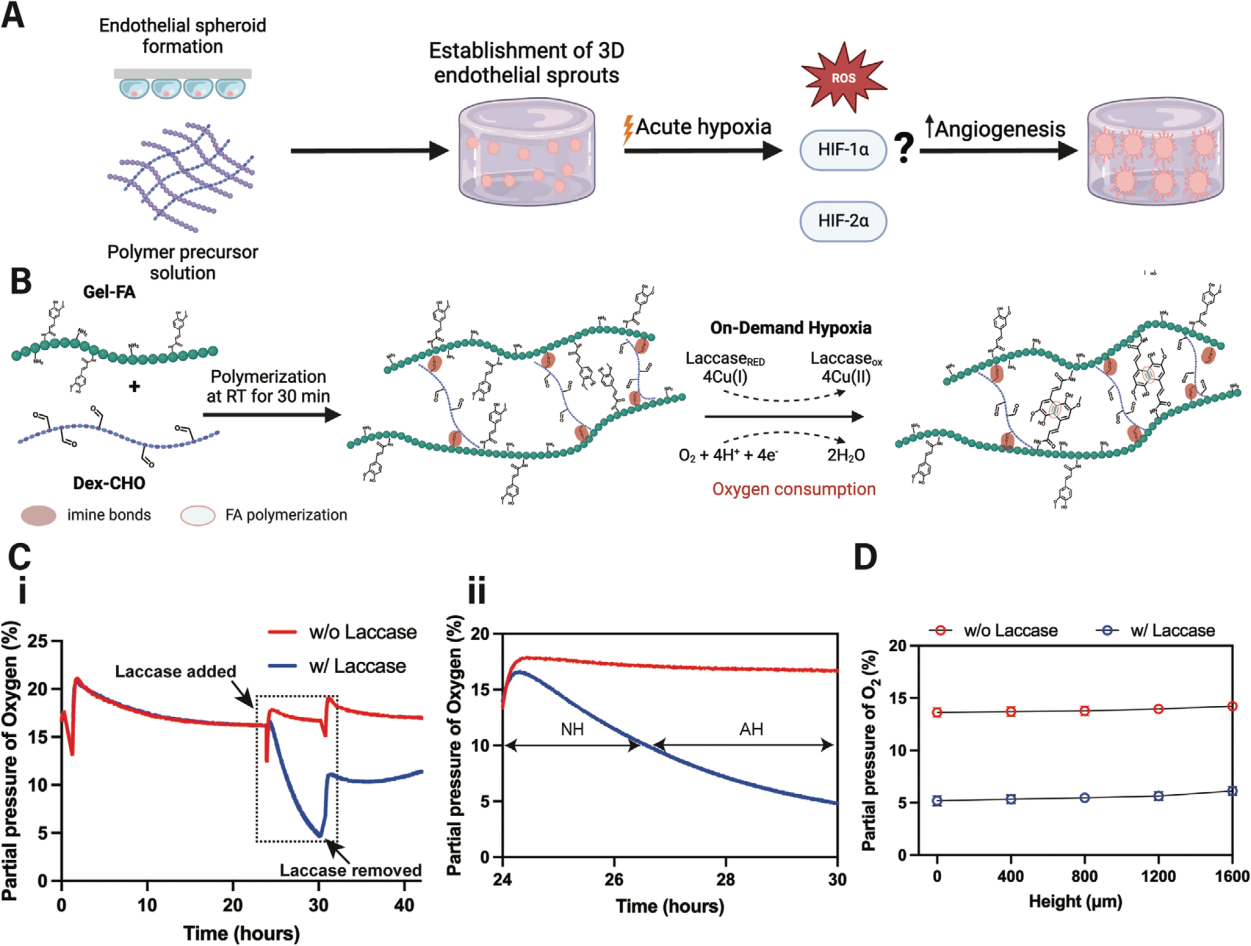
Fabrication of the “on‐demand” hypoxic Gel‐Dex hydrogel. A) Experimental plan to investigate the effect of acute hypoxia on 3D‐established endothelial sprouts. B) Schematic of the Gel‐Dex hydrogel system. C) i‐ Representative noninvasive dissolved O_2_ readings at the bottom of Gel‐Dex (*N* = 3 hydrogels per condition). The arrow indicates the time point at which laccase (2.5 U mL^−1^) was added to the hydrogel system. ii‐ Dissolved O_2_ readings within the highlighted dotted area of i‐. Gel‐Dex remains nonhypoxic (NH) within the first ≈3 h of laccase incubation. The laccase‐mediated O_2_ consumption generates moderate acute hypoxia (AH, 5–10% O_2_) within approximately the next 3 h. Laccase is removed after the total 6‐h incubation period. D) Dissolved O_2_ measurements 6 h after the addition of laccase at different heights within the hydrogel (*N* = 3 hydrogels per condition).

Short‐term exposure to moderate hypoxia, followed by tissue re‐oxygenation, also known as hypoxic preconditioning, has been investigated in vivo following ischemic episodes.^[^ ^1^, ^35^, ^36^ ^]^ Indeed, hypoxic preconditioning in vivo at hypoxia chambers set at 8–10% O_2_ for ≈3 to 4 h has been shown to increase vascular density in the brain^[^ ^21^ ^]^ and myocardium^[^ ^17^ ^]^ following tissue ischemia. Moreover, in adult tissues, such short‐term (i.e. acute) hypoxia impacts the already‐established vascular sprouts rather than individual cells, eliciting an enhanced angiogenic response.^[^ ^17^, ^18^, ^19^, ^24^ ^]^ Angiogenic spheroids have been extensively utilized as an in vitro model to determine angiogenesis mechanisms and drug responses.^[^ ^37^, ^38^, ^39^ ^]^ Thus, we sought to establish a hydrogel system that would support sprouting from endothelial spheroids and, subsequently, introduce acute hypoxia rapidly in the surrounding microenvironment (**Figure** **1**A). In our previous work, we developed gelatin‐based hypoxia‐controllable hydrogels using ferulic acid (FA) and laccase enzyme.^[^ ^30^, ^31^, ^32^, ^33^ ^]^ The laccase‐mediated dimerization of FA groups on gelatin facilitated both polymerization and O_2_ consumption, enabling 3D encapsulation of single cells while inducing hypoxia simultaneously. This allowed us to study the role of hypoxia in new blood vessel formation (vasculogenesis) and the formation of EC clusters using this in vitro platform. However, as this platform initiates hypoxia and gelation simultaneously, it limits the opportunities to study angiogenesis, the formation of new blood vessels from pre‐existing ones.^[^ ^40^ ^]^ To address this, we set to refine the hypoxia hydrogel model to decouple the hypoxia induction from the polymerization process. Here, “on‐demand” hypoxic hydrogels were produced using gelatin‐ferulic (Gel‐FA) and aldehyde‐modified dextran (Dex‐CHO), allowing for temporal, user‐defined modification of the extracellular environment. First, polymerization occurs through reversible imine bonds forming between the amine groups of the Gel‐FA and the aldehyde groups of Dex‐CHO, creating a covalent network. This dynamic hydrogel network provides a suitable microenvironment for in vitro cell culture, effectively mimicking the biomechanical properties of native soft tissues^[^ ^41^, ^42^, ^43^, ^44^ ^]^ and promoting EC growth and sprouting.^[^ ^45^, ^46^ ^]^ The gelatin‐dextran (Gel‐Dex) hydrogels were prepared off‐stoichiometrically, with an aldehyde to amine ratio of ≈1:20 per g of Dex‐CHO and Gel‐FA, respectively (Figure S1A, Supporting Information). Next, hypoxia is induced by the addition of laccase that consumes O_2_ (Figure 1B). The FA functional groups on gelatin were further utilized for the laccase‐catalyzed reaction to create “on‐demand” hypoxia at a final laccase concentration of 2.5 U mL^−1^. FA groups remain stable under in vitro cell culture conditions, allowing for temporal modification of O_2_ levels in the Gel‐Dex system. The incorporation of FA functional groups on gelatin was designed to be 28.6 ± 3.7 µmol g^−1^ to achieve moderate hypoxia (Figure S1A, Supporting Information). To evaluate the O_2_‐controlling capabilities of the Gel‐Dex hydrogel, we performed continuous noninvasive monitoring of the partial pressure of O_2_ at the bottom of the Gel‐Dex (Figure S1B, Supporting Information). The O_2_ time profile shows that Gel‐Dex rapidly achieves hypoxic O_2_ levels (≈5% O_2_) following laccase incubation (Figure 1Ci). Specifically, the O_2_ level drops to ≈10% within the first 3 h, while the laccase‐mediated O_2_ consumption reaches at ≈5% approximately within the next 3 h (Figure 1Cii). These conditions, in which the FA groups of the Gel‐Dex rapidly generate “on‐demand” moderate hypoxic conditions (≈5–10% O_2_) for a total of 3 h, recapitulate the hypoxic preconditioning approach studied in vivo.^[^ ^17^, ^21^ ^]^ Thus, we focus on the 6‐h timepoint to remove the laccase and investigate how rapid, short‐term hypoxia impacts vascular sprouts. The rapid moderate hypoxia (≈5% O_2_) can be sustained for over 10 h in the standard cell culture incubator without removing the laccase solution (Figure S1C, Supporting Information). It is important to note that due to limitations of molecular transport in the 3D matrix, the partial pressure of O_2_ in the nonhypoxic Gel‐Dex (Figure 1C) is lower than the O_2_ levels of a standard cell culture incubator (≈18% O_2_). Nevertheless, this concentration range (≈14–18% O_2_) is typically higher than what is normally found in tissues.^[^ ^47^ ^]^ Therefore, endothelial cells on either end of this O_2_ range are not expected to be affected differently. We further examined the spatial distribution of dissolved O_2_ after the 6‐h incubation with laccase to further characterize the O_2_ profile within the hydrogel. Invasive needle sensor O_2_ measurements were made starting at the bottom of the hydrogel and partial pressure of O_2_ was recorded every 400 µm up to the top of the hydrogel, (Figure S1D, Supporting Information). Invasive O_2_ readings revealed a uniformly distributed dissolved O_2_ profile within Gel‐Dex (Figure 1D). It is worth noting that laccase diffusion can lead to a gradient of laccase concentration across the 3D matrix, which may result in a heterogeneous O_2_ distribution. However, the invasive O_2_ readings throughout Gel‐Dex show that ≈5% O_2_ is achieved, indicating that laccase‐mediated O_2_ consumption and O_2_ diffusion from the cell culture medium occur dynamically during the 6‐h period, ultimately leading to a homogeneous O_2_ profile within the hydrogel. All together, we developed a hydrogel system that rapidly induces moderate hypoxia “on‐demand,” while maintaining a homogeneous O_2_ profile across its 3D structure. To ensure we mimic the acute hypoxia preconditioning in vivo, we removed laccase after 6 h, thus allowing the system to return to nonhypoxic O_2_ levels.

### 2.2 Acute Hypoxia in Gel‐Dex Hydrogels Does not Alter the Hydrogel's Mechanical Properties

**Figure 2:**
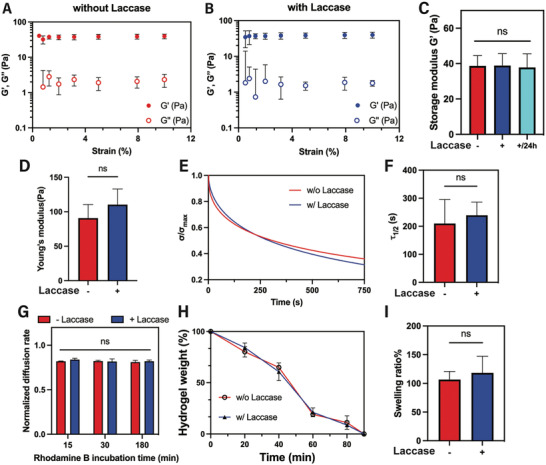
Mechanical characterization of the Gel‐Dex hydrogel system. Amplitude sweeps of Gel‐Dex A) without and B) with the addition of laccase for 6 h. C) Gel‐Dex shear rheology without laccase (−) and with laccase (+) for 6 and 24 h after the 6‐h laccase incubation (*N* = 5 hydrogels per condition). D) AFM measurement of Young's modulus of Gel‐Dex with and without the addition of laccase for 6 h (*N* = 3 hydrogels per condition, ≈13 indentations on average per gel). E) Representative stress‐relaxation curves of Gel‐Dex with and without incubation with laccase. F) Quantification of the timescale at which the stress is relaxed to half of its original value, τ_1/2_, before and after adding laccase (*N* = 3 hydrogels per condition). G) Normalized diffusion rates, H) collagenase IV degradation rate (0.050%), and I) swelling ratio following a 6 hour‐incubation period with laccase compared to hydrogels without laccase (*N* = 3–4 hydrogels per condition). ns = not significant.

To evaluate whether laccase‐mediated creation of hypoxia led to matrix stiffening, hydrogel viscoelasticity was examined using oscillatory shear rheometry and atomic force microscopy (AFM). The storage moduli (G’) of both non‐hypoxic (**Figure** **2**A) and “on‐demand” hypoxic (Figure 2B) Gel‐Dex hydrogels were comparable, with values of 38.68 ± 5.86 and 38.87 ± 6.80 Pa, respectively (Figure 2C), as measured by amplitude sweep. Additionally, we examined the storage modulus of Gel‐Dex 24 h following laccase removal after the 6‐h incubation period, to further assess the potential for matrix stiffening under our current laccase reaction conditions. As shown in Figure 2C, the hydrogel stiffness remained similar to that of both the nonhypoxic and hypoxic hydrogels after 6 h of laccase incubation, suggesting that our O_2_‐consuming reaction mediated by laccase does not significantly alter the matrix stiffness. We further confirmed that the system's Young's modulus did not change following laccase incubation for 6 h (Figure 2D). Stress relaxation measurements were also conducted to examine whether 6 h of laccase incubation changed the stress relaxation profiles of the “on‐demand” hypoxic Gel‐Dex gels compared to their nonhypoxic counterpart. Stress relaxation, the ability of a hydrogel to recover from external deformation, has emerged as a key property that impacts cell behavior.^[^ ^42^, ^45^, ^48^, ^49^ ^]^ Figure 2E shows that the rate of stress relaxation was similar between the non‐hypoxic and “on‐demand” hypoxic gels. Specifically, the time for the initial stress of the Gel‐Dex gels to be relaxed to half its value (Figure S2A, Supporting Information) during a stress relaxation test (τ_1/2_) was comparable, confirming that induction of acute hypoxia does not change the hydrogel's relaxation rate (Figure 2F). We further examined additional physiochemical properties of Gel‐Dex gels including diffusion rate (Figure 2G), proteolytic degradation (Figure 2H), and swelling ratio (Figure 2I), and found no significant change following the 6‐h laccase incubation timeframe. In summary, we show that the introduction of acute hypoxia through the laccase‐mediated reaction does not change the mechanical properties of Gel‐Dex. This allows us to investigate how acute hypoxia affects cellular processes without being influenced by changes in the matrix network.

### 2.3 “On‐Demand” Hypoxic Gel‐Dex Hydrogels Support Endothelial Spheroids

**Figure 3:**
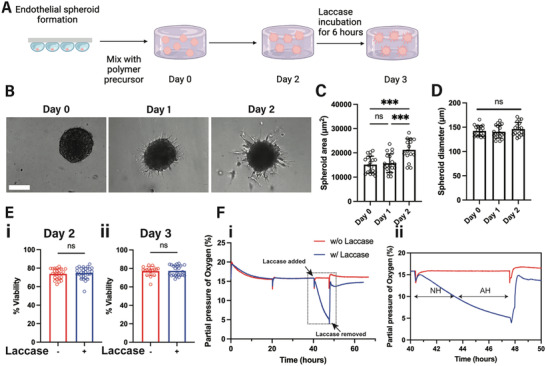
The dynamic hypoxic Gel‐Dex hydrogel supports endothelial spheroid growth. A) Schematic timeline of the spheroid formation, encapsulation, and induction of hypoxia “on‐demand” in Gel‐Dex. B) Representative brightfield images of endothelial spheroids encapsulated in Gel‐Dex. C) Quantification of the spheroid area and D) diameter over a period of 2 days in Gel‐Dex (*N* = 3 biological replicates, n = 15–19 spheroids per condition). E) i‐ Live/dead assay to quantity the viability of endothelial spheroids immediately after the 6 hour‐incubation period with laccase on day 2 and ii‐ on day 3, after removal of laccase on day 2 (*N* = 3 biological replicates, *n* = 17–28 spheroids per condition). F) i‐ Representative noninvasive monitoring of the partial pressure of O_2_ during the overall 3‐day culture period of endothelial spheroids in Gel‐Dex (*N* = 3 biological replicates). The “peaks” that appear in both conditions are due to media changes. Laccase was added in the media on day 2 at the timepoint indicated by the arrow and was removed after 6 h. ii‐ Dissolved O_2_ readings at the bottom of the hydrogel during laccase incubation (highlighted dotted area in i‐). Spheroids were exposed to nonhypoxic (NH) conditions for ≈3 h, followed by exposure to 5–10% O_2_ (acute hypoxia, AH) for ≈3 h. Significance levels were set at: ns = not significant, ***p < 0.001. Scale bar is 100 µm.

After fabricating a system that introduces acute hypoxia “on‐demand” without compromising hydrogel mechanics, we sought to determine whether Gel‐Dex would facilitate the growth of endothelial sprouts. To this end, we generated endothelial spheroids from ECFCs using the hanging droplet method.^[^ ^37^ ^]^ After 24 h, we collected the endothelial spheroids and encapsulated them in Gel‐Dex precursor solution (**Figure** **3**A). Following hydrogel formation, we allowed spheroids to establish sprouts for 2 days (Figure 3B). It is worth noting that angiogenic growth factors, including VEGF and FGF, are needed to facilitate sprout formation in in engineered biomaterials such as ours.^[^ ^50^, ^51^, ^52^, ^53^ ^]^ Nonetheless, we observed an increase in spheroid area (Figure 3C), but not in diameter (Figure 3D) during the culture period. After confirming that Gel‐Dex enables sprouting from endothelial spheroids, we sought to investigate the effect of acute hypoxia on the sprouts. On day 2, we added laccase to the cell culture medium on top of Gel‐Dex for 6 h to generate moderate acute hypoxia in the surrounding microenvironment of the established sprouts. Incubation with laccase for 6 h on day 2 did not compromise cell viability (Figure S3A, Supporting Information) immediately after (Figure 3Ei) or on day 3 (Figure 3Eii). Excess copper and elevated copper‐dependent enzymes have been shown to promote cellular senescence.^[^ ^54^ ^]^ We found that incubation with 2.5 U mL^−1^ laccase in cell media, a multi‐copper oxidase, for 6 h in 2D ECFCs does not elevate p21 expression (Figure S3B, Supporting Information), a well‐established cellular senescence marker.^[^ ^55^, ^56^ ^]^ After confirming that our hydrogel platform is biocompatible, we monitored the dissolved O_2_ levels throughout the 3‐day culture period using noninvasive patch sensors. Similar to the acellular hydrogel, the addition of laccase in the cell culture medium on day 2, resulted in a drop of dissolved O_2_ levels to ≈5% within 6 h (Figure 3Fi‐ii), suggesting that cellular consumption of O_2_ is negligible in our system. Thus, we constructed a biocompatible hydrogel platform that supports 3D sprouting from endothelial spheroids and can introduce acute moderate hypoxia “on‐demand” on the established vascular sprouts.

### 2.4 Acute Hypoxia Induces Extensive Endothelial Sprouting, Migration, and Proliferation

**Figure 4:**
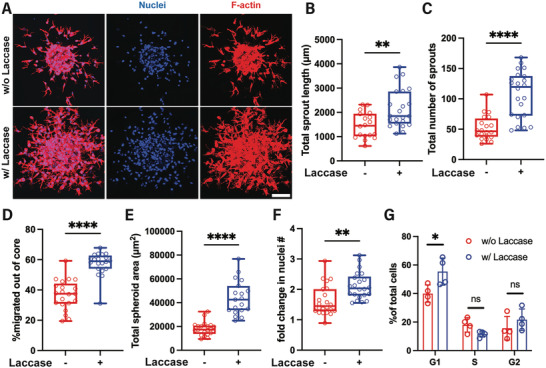
“On‐demand” hypoxia enhances migration and sprouting in endothelial spheroids. A) Day 3 representative images of maximum intensity projection of confocal z‐stack of endothelial spheroids in the Gel‐Dex hydrogel without (top panel) and with (bottom panel) the addition of laccase on day 2 for 6 h. “On‐demand” acute hypoxia increases the B) total sprout length and C) number of sprouts per spheroid. D) Acute hypoxia promotes enhanced cell migration out of the endothelial core compared to nonhypoxic hydrogels. E) The “on‐demand” hypoxic hydrogels Gel‐Dex show a greater area of endothelial spheroids compared to their nonhypoxic counterparts. F) Fold‐change of the number of cell nuclei observed per spheroid on day 3, normalized to the average number of cell nuclei observed on day 2, with (+) and without (−) laccase incubation. (*N* = 3–4 biological replicates, n = 18–22 spheroids per condition). G) Distribution of cells in each phase of the cell cycle with and without laccase incubation (*N* = 4 biological replicates, 5–7 Gel‐Dex hydrogels with ≈30 spheroids each were used per experiment). Scale bar is 100 µm. Significance levels were set at: ns = not significant, *p < 0.05, **p < 0.01, ****p < 0.0001.

After establishing a platform that initially facilitates the formation of endothelial sprouts and, subsequently, induces hypoxia “on‐demand,” we focus on assessing the individual effect of acute hypoxia on sprouting. In vivo studies have shown that short‐term moderate hypoxic exposure promotes angiogenesis upon re‐oxygenation in various tissues including brain^[^ ^21^ ^]^ and myocardium.^[^ ^17^, ^18^, ^19^, ^20^, ^57^ ^]^ To recapitulate this process in vitro, endothelial spheroids encapsulated in Gel‐Dex were incubated with laccase for 6 h on day 2, thus exposing the established sprouts to acute moderate hypoxia (**Figure** **4**A). The next day, we observed an increase in the total length (Figure 4B) and number (Figure 4C) of endothelial sprouts in the “on‐demand” hypoxic hydrogels compared to their nonhypoxic counterparts, indicating that acute hypoxia promotes angiogenesis from established sprouts. Interestingly, the current “on‐demand” strategy also results in increased total sprout length (Figure S4A,B, Supporting Information) but not total sprout number (Figure S4C, Supporting Information) compared to inducing hypoxia immediately upon spheroid encapsulation by directly mixing laccase with Gel‐Dex precursor solution. We postulate that initially establishing sprouts in the 3D matrix and then introducing hypoxia through laccase diffusion creates a more favorable microenvironment, allowing the progression of angiogenesis through rapid cell migration, as opposed to the laccase pre‐mixing approach. Sprouting angiogenesis commences upon EC activation and is essential in both developing and adult tissues.^[^ ^1^ ^]^ Hypoxia stimulates ECs, causing them to migrate from the parent vessel and increasing their proliferation, thus guiding angiogenesis.^[^ ^40^, ^58^, ^59^ ^]^ In accordance with that, we found that the induction of acute hypoxia on day 2 enhanced endothelial cell migration out of the spheroid core (Figure 4D), resulting in an increase in spheroid area (Figure 4E). While exposure to severe hypoxic conditions for prolonged periods of time has been shown to attenuate proliferation and cell cycle progression,^[^ ^60^, ^61^, ^62^ ^]^ short‐term exposure to moderate hypoxia has been shown to promote endothelial cell proliferation.^[^ ^62^, ^63^, ^64^ ^]^ On day 3, we observed an increase in the total number of cells (Figure 4F), following a 6‐h laccase incubation on day 2, suggesting that acute exposure to moderate hypoxia enhances proliferation. In addition, cell cycle analysis using flow cytometry showed a greater percentage of cells distributed to G1 phase in the “on‐demand” hypoxic hydrogel compared to nonhypoxic controls, indicating that short‐term induction of moderate hypoxia facilitates cell cycle progression (Figure 4G). Taken together, we show that rapid induction of short‐term moderate hypoxia enhances endothelial cell migration from pre‐existing sprouts, causing them to proliferate more and form new sprouts, thus promoting angiogenesis. The “on‐demand” hypoxic hydrogel is the first in vitro platform that demonstrates how acute hypoxia alone guides angiogenesis.

### 2.5 ROS, Independently of HIFs, Regulate Angiogenesis in Acute Hypoxia

**Figure 5:**
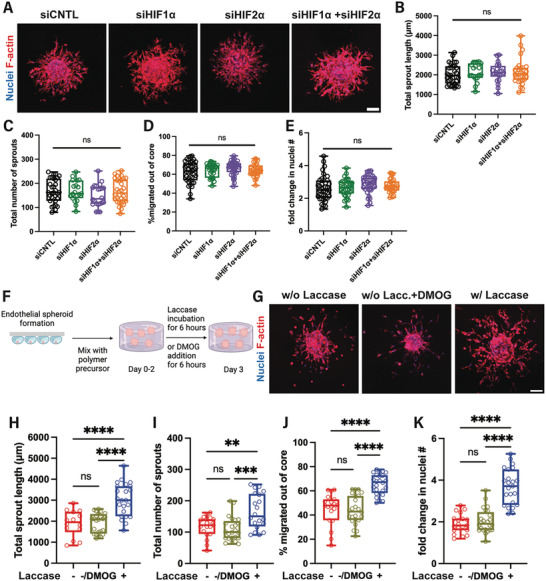
The enhanced endothelial sprouting under acute hypoxia is independent of HIFs. A) Day 3 representative images of endothelial spheroids in Gel‐Dex, following 6 hour‐laccase incubation on day 2 and, HIF knockdown (siHIF1α, siHIF2α, and siHIF1α+siHIF2α) compared to siRNA control (siCNTL). Quantification of the B) total length and C) number of endothelial sprouts, on day 3 following a 6‐h laccase incubation on day 2. D) siRNA knockdown of HIFs does not change cell migration or E) total cell nuclei number on day 3, following incubation with laccase for 6 h on day 2. (*N* = 3 biological replicates, n = 19–38 spheroids per condition). F) Experimental timeline for the pharmacological induction of HIF accumulation in Gel‐Dex under nonhypoxic conditions. G) Day 3 representative images of maximum intensity projection of confocal z‐stack of endothelial spheroids in the Gel‐Dex hydrogel show that incubation with DMOG does not induce increased angiogenic sprouting in nonhypoxic hydrogels. Quantification of the H) total sprout length, and I) total number of sprouts per spheroid with (+) without (–) laccase incubation for 6 h on day 2. J) Inhibition of HIF degradation with DMOG does not lead to enhanced cell migration out of the spheroid core under nonhypoxic conditions or K) increased cell nuclei number per spheroid on day 3. (*N* = 3 biological replicates, n = 14–24 spheroids per condition). Scale bars are 100 µm. Significance levels were set at: ns = not significant, **p < 0.01, ***p < 0.001, ****p < 0.0001.

**Figure 6:**
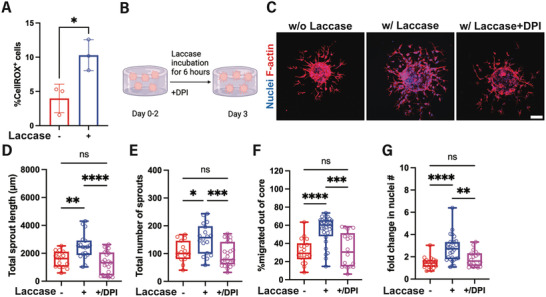
The enhanced angiogenesis observed under acutely induced moderate hypoxia depends on ROS. A) Introduction of moderate hypoxia “on‐demand” results in increased ROS production following a 6‐h incubation with laccase. B) Experimental timeline for the investigation of ROS‐mediated sprouting. C) Day 3 representative images of maximum intensity projection of confocal z‐stack of endothelial spheroids in the Gel‐Dex hydrogel show that inhibition of ROS attenuates the increased angiogenic sprouting under hypoxia. Quantification of the D) total sprout length, and E) total number of sprouts per spheroid with (+) and without (–) laccase incubation for 6 h on day 2. F) Small‐molecule inhibition of ROS impedes cell migration out of the spheroid core under hypoxia and G) fold‐change quantification of the total cell nuclei number observed per spheroid on day 3, normalized to the average number of cell nuclei observed on day 2, with and without laccase incubation. (*N* = 3–4 biological replicates, n = 15–28 spheroids per condition). Scale bars are 100 µm. Significance levels were set at: ns = not significant, *p < 0.05, **p < 0.01, ***p < 0.001, ****p < 0.0001.

Next, we sought to investigate the underlying mechanism of the enhanced sprouting angiogenesis observed following acute induction of moderate hypoxia. Hypoxia‐inducible factors (HIF) are the primary regulators of cellular adaptation to hypoxia by regulating the transcription of a large set of genes, including those involved in cell survival, angiogenesis, and glycolysis. The HIF1α and HIF2α subunits are only expressed during hypoxia and are the two transcription factors involved in the cellular response regulating matrix degradation, migration, and proliferation.^[^ ^1^, ^2^ ^]^ To assess whether HIFs mediate the increased angiogenic response we observed under acute hypoxia, we knocked down HIF1α and/or HIF2α using small interfering RNA (siRNA) prior to spheroid formation. We confirmed gene knockdown 24 h post‐transfection (Figure S5A,B, Supporting Information) and proceeded to spheroid formation and encapsulation in Gel‐Dex polymer precursor solution. Following laccase incubation for 6 h on day 2, we found that silencing of HIF1α and/or HIF2α (**Figure** **5**A) did not change the total length (Figure 5B) or number (Figure 5C) of sprouts. In addition, we did not observe any changes in the percentage of cells migrating out of the endothelial spheroid core (Figure 5D) or the total number of cell nuclei on day 3 (Figure 5E). We further utilized dimethyloxalylglycine (DMOG) to prevent HIF degradation under nonhypoxic conditions (Figure 5F). DMOG is a potent prolyl‐4‐hydroxylase (PHD) inhibitor, stabilizing HIFs under nonhypoxic conditions and has been extensively used in vitro and in preclinical models.^[^ ^65^, ^66^ ^]^ We found that DMOG treatment (Figure 5G) did not result in increased total sprout length (Figure 5H) or number (Figure 5I) in the nonhypoxic hydrogels. Similarly, in the DMOG‐treated hydrogels, we observed comparable cell migration (Figure 5J) and nuclei number (Figure 5K) to that of their untreated nonhypoxic counterparts. Our findings indicate that neither HIF1α nor HIF2α regulates the enhanced sprouting we observed under acute hypoxia. We hypothesize this is due to the moderate hypoxic levels (≈5% O_2_) achieved by our platform. Indeed, HIF1α is proteolytically degraded when oxygen levels vary between 6% and atmospheric level, while its stability rises exponentially as oxygen levels approach 0.5–1%.^[^ ^67^ ^]^ ECs exposed to O_2_ <1% have shown peak expression of HIF1α within 2–4 h, while prolonged exposure (6–20 h) increases HIF2α expression while simultaneously decreasing HIF1α.^[^ ^68^ ^]^ Additional modes of HIF‐independent cellular response under hypoxia have also been documented.^[^ ^69^, ^70^, ^71^ ^]^ ROS produced at the mitochondrial complex III are involved in most investigated O_2_‐sensing mechanisms, including pathways beyond the canonical HIF response.^[^ ^2^, ^70^ ^]^ Mitochondrial ROS start to produce at ≈5% O_2_^[^ ^2^ ^]^ and have been proposed to orchestrate the initial response to acute hypoxia.^[^ ^72^, ^73^, ^74^ ^]^ Indeed, we found an increase in ROS production following a 6‐h incubation with laccase, compared to nonhypoxic hydrogels (**Figure** **6**A). To assess whether the production of ROS is responsible for the pro‐angiogenic response observed under our moderate hypoxic conditions, we utilized a potent ROS inhibitor, diphenyleneiodonium chloride (DPI), during the 6‐h laccase incubation period (Figure 6B). Inhibition of ROS production under short‐term hypoxia (Figure 6C) reduced endothelial sprouting (Figure 6D,E), as well as cell migration (Figure 6F) and total cell nuclei (Figure 6G). Taken together, using the on‐demand hypoxic Gel‐Dex we demonstrate that short‐term exposure of established sprouts to moderate hypoxia (≈5–10% O_2_) promotes angiogenesis in a ROS‐mediated manner, independently of HIFs.

### 2.6 Matrix Degradation Facilitates Angiogenesis Following Acute Hypoxia

**Figure 7:**
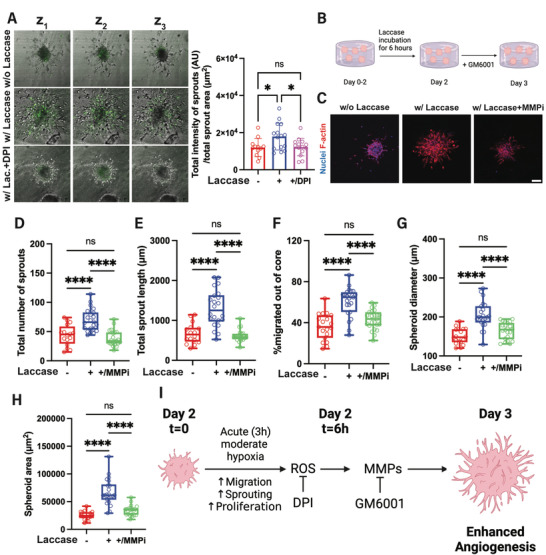
Matrix metalloproteases facilitate increased endothelial sprouting under rapid hypoxia. A) *Left‐* Fluorescent microscopy images overlay light microscopy images show DQ gelatin fluorescence (green) in the proximity of the endothelial sprouts. Representative brightfield images approximately at the bottom (z_1_), middle (z_2_), and top (z_3_) of each spheroid. *Right‐* corresponding quantification (*N* = 3 biological replicates, n = 12–15 spheroids per condition). B) Experimental timeline for the investigation of MMP‐mediated sprouting under hypoxia. C) Day 3 representative images of maximum intensity projection of confocal z‐stack of endothelial spheroids in the Gel‐Dex hydrogel show that broad inhibition of MMPs negates the increased angiogenic sprouting under hypoxia. Small molecule inhibition of MMPs attenuates the enhanced angiogenic response under hypoxia. Quantification of the D) total sprout number, E) total sprout length, and F) cell migration out of the endothelial spheroid core. Quantification of spheroid G) diameter and H) area under induced hypoxia with and without MMP inhibitor. (*N* = 3 biological replicates, n = 15–28 spheroids per condition). I) Proposed mechanism for the enhanced angiogenic response of established endothelial sprouts under the induction of acute 3D hypoxia. Scale bar is 100 µm. Significance levels were set at: ns = not significant, *p < 0.05, ****p < 0.0001.

Proteolytic degradation plays a key role in angiogenesis. Following hypoxia, ECs upregulate the expression of matrix metalloproteinases (MMPs) through HIF and/or ROS‐dependent mechanisms^[^ ^75^, ^76^, ^77^, ^78^ ^]^ and degrade the surrounding matrix, which allows for enhanced angiogenic sprouting under hypoxia. There are numerous upstream regulators of MMPs, but most notably, ROS have been shown to rapidly induce MMP production.^[^ ^79^ ^]^ MMP gene expression, including MMP‐1,^[^ ^80^ ^]^ MMP‐2,^[^ ^81^ ^]^ MMP‐7,^[^ ^82^ ^]^ and MMP‐9^[^ ^83^ ^]^ can be modulated by ROS through interactions with thiol groups.^[^ ^81^, ^84^ ^]^ To investigate proteolytic degradation under acute hypoxia in Gel‐Dex, we used DQ‐gelatin, which fluoresces as it is degraded by proteolysis and thus can identify a broad range of MMPs.^[^ ^32^, ^34^, ^85^ ^]^ On day 3, we observed an increase in fluorescence intensity of the DQ substrate in the proximity of the endothelial sprouts area following laccase addition for 6 h which is attenuated upon DPI treatment (**Figure** **7**A). This indicates that proteolytic degradation of the ECM in the vicinity of established sprouts is ROS‐dependent and facilitates the progression of angiogenesis following acute hypoxia. We further observed decreased fluorescence intensity in the media of the “on‐demand” hypoxic hydrogels following DPI treatment (Figure S6, Supporting Information), suggesting an overall suppression of MMP activity in those conditions. In our system, we found that ROS plays a crucial role in the generation of new sprouts under acute moderate hypoxia. Our findings indicate that MMP expression is regulated downstream of ROS activity, we thus postulated that MMP inhibition following moderate hypoxic exposure would negate the enhanced angiogenic response. To confirm this, we used GM6001, a broad‐spectrum small molecule MMP inhibitor, on day 2 following laccase incubation (Figure 7B). We found that pharmacological inhibition of MMPs following induction of acute hypoxia (Figure 7C) decreased the total number (Figure 7D) and length of endothelial sprouts (Figure 7E). In addition, inhibiting matrix degradation attenuated cell migration (Figure 7F), yielding spheroid diameter (Figure 7G) and area (Figure 7H) comparable to the non‐hypoxic conditions. To summarize, we showed that when established endothelial sprouts are acutely exposed to moderate hypoxic conditions, their individual endothelial cells become more migratory and proliferative. Simultaneously, upregulation of ROS activates MMPs, allowing for efficient ECM degradation, thus enhancing angiogenesis from the established endothelial sprouts (Figure 7I).

## 3 Conclusion

Here, we report the fabrication of the Gel‐Dex hydrogel system that decouples polymerization from O_2_ consumption allowing the introduction of acute hypoxia “on‐demand” and the study of its effect on endothelial sprouts. Expanding on our previous work, we generated a platform that rapidly generates short‐term hypoxia in a Gel‐Dex hydrogel and facilitates real‐time monitoring of the moderately hypoxic O_2_ levels encountered by vascular sprouts in their surrounding microenvironment in vivo. Introduction of laccase in the cell culture medium and its subsequent diffusion through Gel‐Dex, acutely reduced the partial pressure of O_2_ to moderately hypoxic levels (≈10%O_2_), facilitating the exposure of established vascular sprouts to acute hypoxia (≈5–10% O_2_) for ≈3 h. Using this platform, we showed that short‐term moderate hypoxia enhanced endothelial cell migration and proliferation, promoting angiogenesis from pre‐existing 3D vascular sprouts. Using shear rheometry and AFM, we showed that the laccase‐mediated oxygen‐consuming reaction did not impact the system's mechanical properties while successfully establishing short‐term moderately hypoxic conditions. Therefore, Gel‐Dex can be utilized to effectively recapitulate the enhanced angiogenic response observed following hypoxic preconditioning.

Ischemia has been shown to enhance EC proliferation and overall angiogenesis at the border of infarction in a HIF‐mediated manner.^[^ ^86^, ^87^ ^]^ HIF‐1α is expressed rapidly following ischemia,^[^ ^88^ ^]^ promoting angiogenesis, while following prolonged hypoxic exposure, HIF‐2α stabilizes the newly formed vessels.^[^ ^89^ ^]^ Using siRNA inhibition, we found that knocking down HIF1α or HIF2α, individually or in combination, did not attenuate the increased angiogenesis under short‐term hypoxia, indicating that the underlying mechanism is independent of HIFs, possibly due to the moderate hypoxic levels achieved by our platform (≈5% O_2_). ROS are primarily produced by the mitochondrial complex III^[^ ^73^, ^74^, ^90^, ^91^ ^]^ and possess a multitude of signaling roles in vascular endothelium.^[^ ^92^ ^]^ The exact O_2_ level at which they start to produce is not clearly defined. However, it has been reported that cells exposed to moderate hypoxic levels^[^ ^2^, ^93^ ^]^ increased their ROS production. We found that inhibiting ROS production in moderate and acute hypoxic conditions impeded EC migration and proliferation while reducing the total number and length of endothelial sprouts. Taken together, these findings suggest that the enhanced angiogenic response we observed under moderate short‐term hypoxia is orchestrated by ROS, independently of HIFs. ECs upregulate MMP expression, promoting ECM degradation, cell migration, and new sprout formation.^[^ ^1^ ^]^ We found increased MMP activity under moderate short‐term hypoxia and further demonstrated that broad pharmacological inhibition of MMPs following moderate hypoxic exposure diminished angiogenesis, thus confirming their role in modulating angiogenesis under short‐term hypoxia. Future work will focus on delineating ROS‐dependent protein targets that contribute to angiogenesis under short‐term hypoxia. To our knowledge, Gel‐Dex is the first hydrogel to allow controllable acute O_2_ regulation in the vicinity of established endothelial sprouts, scrutinizing its role in angiogenesis. Our in vitro platform can set the foundation for studying hypoxic preconditioning as a potential therapeutic approach for ischemic episodes.

## 4 Experimental Section

### Materials

Gelatin (Gel, from porcine skin gel strength 300, Type A; G2500), trans‐ferulic acid (FA; 128708), N‐(3‐dimethylaminopropyl)‐N′‐ethylcarbodiimide hydrochloride (EDC; E6383), N‐hydroxysuccinimide (NHS; 56480), dimethyl sulfoxide (DMSO; 276855), and bovine serum albumin (BSA; A3059), Deuterium oxide (450510‐10 × 0.75ML), Diphenyleneiodonium chloride; DPI (D2926), Dextran (Mn = 110,000) (09184), Methyl cellulose (M7027‐100G), Sodium Periodate (S1878‐25G), Rhodamine B (R6626‐25G), Triton X‐100 (T8787), and DMOG (D3695‐10MG) were purchased from Sigma‐Aldrich (St. Louis, MO) and used as obtained without purification. Laccase (1560 U mL^−1^ from *Coriolopsis gallica*) was purchased from the laboratory of Dr. Rafael Vazquez‐Duhalt, Centro de Nanociencas y Nanotechología UNAM. Dulbecco's phosphate‐buffered saline (DPBS; 14190250), TryplE (12604021), Halt Protease and phosphatase inhibitor cocktail (ProtInh) (100×) and EDTA (78440), Trizol reagent (15596018), Ambion Nuclease‐Free Water (AM9906), TaqMan Gene Expression assays (4453320) (HIF1A; Hs00153153_m1, EPAS1; Hs01026149_m, formaldehyde 37% by weight (formaldehyde; F79), Alexa Fluor 546 phalloidin (phalloidin; A22283), Alexa Fluor 635 phalloidin (phalloidin; A34054), Live/Dead kit (L3224), 4′,6‐diamidine‐2′‐phenylindole dihydrochloride (DAPI; D1306), SuperSignal West Pico PLUS Chemluminescent Substrate (34577), and DQ Gelatin from Pig Skin Fluorescein Conjugate (DQ‐gelatin D12054), were all purchased from Thermo Fisher Scientific (Waltham, MA). P21 Rabbit mAb (#2947) and Anti‐rabbit IgG HRP‐linked (7074) were purchased from Cell Signaling Technology (Danvers, MA). Dialysis membranes (molecular mass cutoff  =  6–8 kDa) (132645) were purchased from Repligen (Boston, MA). Endothelial growth media‐2 (EGM2; CC‐3162) was purchased from Lonza (Walkersville, MD) and supplemented with additional characterized HyClone fetal bovine serum (FBS) from Cytiva (SH30071.03HI) (Marlborough, MA) and used to culture ECFCs (provided by M. Yoder, Indiana University School of Medicine) on collagen I, rat tail (Col I; 354236) from Corning (Corning, NY) coated cell culture plates. Recombinant Human VEGF 165 (100‐20‐50UG) and Human FGF‐basic (154 a.a.) (100‐18B‐100UG) were purchased from PeproTech (Cranbury, NJ). Cell Staining Buffer (420201) and Propidium Iodide Solution (421301) were purchased from Biolegend (San Diego, CA). SMARTpool:siGENOME Human EPAS1 siRNA 5nmol (M‐004814‐01‐0005), SMARTpool:siGENOME Human HIF1A siRNA 5 nmol (M‐004018‐05‐0005), siGENOME Non‐Targeting Control siRNA 1 5nmol (D‐001210‐01‐05), 5× siRNA Buffer, 100 mL (B‐002000‐UB‐100 ), Molecular Grade RNase‐free water (B‐003000‐WB‐100 ), and Dharmafect1 Transfection Reagent (T‐2002‐02) were purchased from Horizon Discovery (Cambridge, UK). Ilomastat (GM6001; HY‐15768) was purchased MedChemExpress (Monmouth Junction, NJ). RNeasy Mini Kit (74106) was purchased from QIAGEN (Hilden, Germany). Collagenase Type IV (#07426) was purchased from STEMCELL Technologies (Vancouver, Canada). GoScript Reverse Transcriptase kit (A5001) was purchased from Promega. Thick Blot Filter paper (1703932), Tris/Glycine/SDS Electrophoresis Buffer 10X (1610772), 4–20% Mini‐PROTEAN TGX™ Precast Protein Gels, 10‐well, 50 µL (4561094), were purchased from Bio‐Rad (Hercules, CA). CellROX Green Flow Cytometry Assay Kit (C10492) was purchased from Invitrogen (Waltham, MA).

### Synthesis of Gelatin‐Ferulic Acid

Gelatin ferulic acid (Gel‐FA) was synthesized using EDC and NHS as coupling reagents. A mixture of DMSO and DI water (1:1 volume ratio) was prepared as a solvent. Gelatin (1.0 g) was dissolved in 50 mL of the solvent at 40 °C. FA (0.39 g, 2.0 mmol) was dissolved in 20 mL of the solvent and reacted with EDC (0.92 g, 4.8 mmol) at room temperature for 15 min and then with NHS (0.64 g, 5.6 mmol) at room temperature for 15 min to activate the terminal carboxyl groups of FA (carboxyl/EDC/NHS = 1:2.4:2.8). The activated solution was then added to the gelatin solution, and the conjugative reaction was conducted at 40 °C for 24 h. Following completion of the reaction, the solution was dialyzed against DI water for 5 days (molecular mass cutoff = 6–8 kDa) and then lyophilized for 3 days.

### Synthesis of Multi‐Aldehyde Modified Dextran

1.0 g, 6.2 mmol) of dextran was dissolved in 100 mL of DI water, and then sodium periodate (1.0 g, 4.6 mmol) in 1 mL of distilled water was added dropwise. The solution was stirred in the dark for 20 min. The oxidation reaction was terminated by adding 1.0 mL ethylene glycol and stirring for an additional 30 min. The mixture was then dialyzed (molecular mass cutoff = 6–8 kDa) against distilled water for a week with the water changed every day, followed by lyophilization to obtain Dex‐CHO.

### Characterization of Hydrogel Precursor Solutions

Gel‐FA polymer solution was prepared at 10 mg mL^−1^ of D_2_O and its chemical structure was confirmed using ^1^H NMR spectrometer (500 MHz Agilent/Varian VNMRS), as previously described.^[^ ^30^ ^]^ The degree of substitution of FA was measured using a UV–vis spectrometer. Gel‐FA (10 mg) was dissolved in 1 mL of DMSO:DI water (1:1) solution, and the absorbance was measured at 320 nm. The concentration of the conjugated FA was calculated 32.18 ± 5.02 mmol g^−1^ based on calibration curve obtained by measuring the absorbance of solutions of known FA concentration. The degree of substitution of aldehyde groups on Dex‐CHO was determined using commercial fluorometric aldehyde quantification kit (Abcam, ab138882). The availability of primary amines on Gel‐FA was measured following 15‐min reaction with fluorescamine with a fluorescence plate reader (Agilent Synergy H1, Agilent, Santa Clara, CA) at excitation/emission 380/470 nm. Gel‐FA solution was compared to a calibration curve of glycine with known concentrations.

### Preparation of Gel‐Dex “On‐Demand” Hypoxic Hydrogels

Stock hydrogel precursor solutions (Gel‐FA and Dex‐CHO) were prepared in DPBS at a concentration of 8 wt%. The volume of each hydrogel is 65 µL and is composed of Gel‐FA at 4 wt% final concentration and Dex‐CHO at 0.5 wt% final concentration, with the remaining volume being cell culture media. The Gel‐Dex precursor solution was mixed by pipetting all components in a 1.5 mL vial. The mixture was added at 65 µL per hydrogel at a 96‐well plate and was allowed to polymerize for 30 min at room temperature. Following hydrogel formation, 200 µL of cell culture media is added on top of each hydrogel. At the indicated timepoints, laccase was mixed with cell culture media at a final concentration of 2.5 U mL^−1^, passed through a 0.22 µm filter and added on top of each hydrogel, after removing existing media. Following a 6‐h incubation period, the laccase‐containing media was removed and replaced with fresh cell culture media.

### Dissolved Oxygen Measurements

Dissolved O_2_ (DO) was measured noninvasively in both acellular and spheroid‐encapsulated hydrogels at the bottom of hydrogels using commercially available sensor patches (Oxygen Sensor Spot; SP‐Pst3‐NAU‐D5‐YOP) and a multichannel fiber‐optic oxygen meter (OXY‐4 mini) from PreSens (Regensburg, Germany). O_2_ patch sensors were calibrated with manufacturer‐provided calibration values. To measure O_2_ levels at the bottom of hydrogels, the patch sensors were initially immobilized in each well of a 96‐well plate and hydrogels were added on top of the sensors (see Figure S1B, Supporting Information). All experiments were conducted in a controlled environment at 37 °C and 5% CO_2_ in a standard incubator.

DO was measured invasively in acellular hydrogels using commercially available needle‐type oxygen microsensors (NTH‐PSt1‐L5‐TF‐NS40/0.8‐OIW‐EXT0) and a microfiber transmitter (Microx TX3). Sensors were calibrated with manufacturer‐provided calibration values and measurements were conducted at room temperature. Gel‐Dex hydrogels were prepared in a 96‐well plate at a final volume of 65 µL. O_2_ sensors were precisely controlled using a Manual Micromanipulator MM (PreSens). Starting at the bottom of the hydrogel, measurements were recorded every 400 µm within the hydrogel up to the top of the gel (1600 µm) (see Figure S1D, Supporting Information).

### Diffusion Rate

Gel‐Dex hydrogels were prepared as described and allowed to swell overnight in DPBS. To ensure that the addition of laccase does not alter the diffusion properties, Gel‐Dex hydrogels were incubated with cell culture media with laccase for 6 h (2.5 U mL^−1^) or cell culture media without laccase. Rhodamine B (1.0 mg L^−1^ in DPBS) was added on top of each hydrogel at 37 °C and taken out at selected timepoints. Absorbance of each sample was measured using a microplate reader at a wavelength of 554 nm. Diffusion rates were obtained by normalizing each measurement to absorbance readings of rhodamine B solution.

### Swelling Ratio

Hydrogel precursor solutions were added on top of pre‐weighted individual cell culture inserts. Following polymerization, hydrogels were initially allowed to swell overnight in DPBS. To ensure that the addition of laccase does not alter the swelling ratio, Gel‐Dex hydrogels were incubated with cell culture media with laccase for 6 h (2.5 U mL^−1^) or cell culture media without laccase. After 6 h, the solutions were removed and the weight of the swollen hydrogel was measured. The dry hydrogel weight was obtained after overnight incubation at 60 °C. Swelling ratio (%) was calculated as:

(1) $$Swelling\;Ratio\;\left(\%\right)=\frac{W_{s}-W_{D}}{W_{D}}\times \;100$$

where *W_S_* and *W_D_* is the weight of the swollen and dry hydrogel, respectively.

### Collagenase Degradation

Gel‐Dex hydrogels were prepared in pre‐weighted 1.5 mL tubes. Following polymerization, hydrogels were initially allowed to swell overnight in DPBS. To ensure that the addition of laccase does not alter the proteolytic degradation of Gel‐Dex, hydrogels were incubated with cell culture media with laccase for 6 h (2.5 U mL^−1^) or cell culture media without laccase. After 6 h, the solutions were removed and the weight of the swollen hydrogel was measured. Hydrogels were incubated in 500 µL of 0.050 wt% collagenase IV at 37 °C. The collagenase solution was removed from the microtubes at the pre‐determined times and the weight of the degraded hydrogels was measured. Fresh solution was added into each tube after weighing. The degradation rate was then calculated as:

(2) $$Degradation\;rate\;\left(\%\right)=\frac{W_{d}}{W_{i}}\times 100\;$$

where W_i_ and W_d_ is the weight of the initial and degraded hydrogel, respectively.

### Shear Rheology

Hydrogels were prepared as discs in an 8‐mm mold and were allowed to swell prior to measurements. Bulk stiffness was measured using an HR‐20 Discovery Hybrid Rheometer (TA Instruments) equipped with an 8‐mm cross‐hatched parallel plate at 37 °C. Storage modulus *G*' and loss modulus G’’ were monitored at a 0.5%–10% strain range, and a fixed frequency of 0.1 Hz. Axial force was kept at 0.02–0.03 N during measurements. In addition, stress relaxation measurements of Gel‐Dex with and without laccase incubation were performed by time sweep tests at a constant initial strain of 10%. The corresponding stress relaxation curves were then normalized to their initial value and fitted to a stretched exponential function as shown in equation (3) as previously reported:^[^ ^94^ ^]^

(3) $$\frac{\sigma }{\sigma_{0}}=e^{-{\left(\frac{t}{t_{k}}\right)}^{\beta }}\;$$

where *σ/σ_0_* is the normalized stress, τ_k_ is the fitted time constant and β (0 < β < 1) is the stretching exponent (Figure S2A, Supporting Information). The experiment time is t and the relaxation rate, *τ_1/2_*, was quantified as the time for the initial stress to half of its original value. Curve fitting was performed in MATLAB.

### AFM

Hydrogels were prepared as discs in an 8 mm mold and were allowed to swell prior to measurements. Swelled hydrogels were adhered inside a petri dish and submerged in PBS to facilitate measurements. Young's Modulus was measured using a Nanowizard V BioScience (Bruker) equipped with SAA‐SPH‐10UM probes with a setpoint of 0.2 nN. Indentation tests were performed near the center of each hydrogel. The Young's Modulus was calculated by fitting the force‐displacement curves with the Hertz model with an assumed Poisson's ratio of 0.5.

### Cell Culture

All cells were cultured using standard, humidified cell culture incubators at 37 °C and 5% CO_2_. ECFCs (Yoder Lab, Indiana University School of Medicine) were cultured in EGM2 (Lonza) prepared according to the manufacturer's instructions with an additional 10% HyClone FBS, on standard tissue culture plates coated with type I collagen (Corning).

### Spheroid Encapsulation in Gel‐Dex Hydrogels

Cells were grown to confluence and then detached by TryplE. Cells were counted and resuspended at a density of 50 000 cells mL^−1^ in EGM2 containing 20% methylcellulose. Hanging drops were formed by pipetting 20 µL droplets onto the top of a petri dish and inverting. Droplets were incubated with DPBS overnight. After 24 h, spheroids were collected and embedded in Gel‐Dex precursor solution. ≈30 spheroids per hydrogel were initially mixed with EGM2 media supplemented with 50 ng mL^−1^ VEGF and 25 ng mL^−1^ FGF. Gel‐FA and Dex‐CHO precursor solutions were added at final concentrations of 4 wt% and 0.5 wt%, respectively. The spheroid embedded Gel‐Dex precursor mixture was pipetted to 96‐well plate at 65 µL per hydrogel and was allowed to polymerize at room temperature for 30 min. Following hydrogel formation, 200 µL of EGM2 media supplemented with 50 ng mL^−1^ VEGF and 25 ng mL^−1^ FGF was added on top of each hydrogel, and the plate was put in a standard cell culture incubator (37 °C and 5% CO_2_). Media was replaced daily. Brightfield images were captured daily to monitor spheroid morphology using an Olympus IX50 (Olympus; Center Valley, PA) and analyzed with ImageJ software (public domain).

### Immunostaining

On day 3, ECFCs spheroids within Gel‐Dex hydrogels were fixed with 3.7% formaldehyde for 30 min at room temperature. Hydrogels were then washed thrice with DPBS with 10 min in between each wash. Encapsulated ECFC spheroids were incubated for 30 min in 5% BSA, 0.5% Triton X‐100 and 0.05% Tween‐20 in DPBS solution. Spheroids were stained with phalloidin (1:400) overnight at 4 °C using the same solution as the diluent. Hydrogels were then washed with DPBS thrice with 10 min in between each wash. Last, hydrogels were incubated in DAPI solution (1:500) for 30 min at room temperature and then washed with DPBS thrice with 10 min in between.

### Cell Viability

ECFC spheroids were imaged using confocal microscopy (Eclipse Ti2; Nikon). Cell viability was assessed at indicated timepoints using Calcein‐AM/ethidium homodimer‐1 stains (Live/Dead kit, ThermoFisher) and analyzed using the Imaris Sports plugin (Imaris Version 9.6, Bitplane).

### Spheroid Analysis

ECFC spheroids were imaged using confocal microscopy (Eclipse Ti2; Nikon). For spheroid analysis, z stacks of about 150 µm were taken to fully capture the entire 3D spheroid (Imaris Version 9.6, Bitplane). The total number of cells in each spheroid was estimated using the Imaris Spots plugin. An average nuclei number count for day 2 spheroids was used to determine the fold change in the nuclei number on day 3. To estimate the number of cells within the spheroid core, a region of interest (ROI; 160µm × 160µm × 160µm) was drawn at the center of each spheroid and the total number of nuclei within the ROI was counted using the Imaris Spots plugin. The %cells migrated outside of the spheroid core was determined as follows:

(4) $$\%cells\;migrated\;out\;of\;core=\frac{total\;nuclei\;count-nuclei\;within\;ROI}{total\;nuclei\;count}$$

The total number and total sprout length of each spheroid were estimated using the AQuTAS MATLAB algorithm.^[^ ^95^ ^]^ The total number of sprouts was calculated as the sum of associated and non‐associated sprouts for each spheroid, while the total sprout length in µm for each spheroid, the pixel size of the individual confocal images was used.

### Cell Cycle Flow Cytometry

On day 3, spheroid‐embedded Gel‐Dex hydrogels were incubated for 30 min in collagenase IV solution (2.0 mg mL^−1^ in cell culture media) at 37 °C. After hydrogel dissolution, the spheroids from 6 wells for each condition (with and without laccase incubation on day 2) were combined into a single 1.5 mL microfuge tube and spun down at 500 × *g* for 10min. After removing the supernatant by aspirating, the cell pellet for each condition was resuspended in 500 µL of TryplE and incubated at 37 °C for 10 min to dissociate the spheroids into single cells. The solution was spun down again at 500 × g for 10min and the supernatant was removed by aspirating. The single cells were fixed in 70% ice‐cold sterile ethanol and left at 4 °C overnight. The following day, the fixed cell solution was spun down at 10g for 5 mins and the supernatant ethanol was removed by aspirating. The cell pellet for each condition was resuspended in 50 µL RNAse (10 µg µL^−1^) to reduce background stain. Following this, 425 µL of cell staining buffer (Biolegend) and 25 µL of PI staining reagent (Biolegend) was added to the cell pellet and incubated for 4 h at 4 °C in the dark. For the identification of CellROX^+^ cells, spheroids were treated similarly with collagenase IV solution and spun down at 500 × g for 10 min. Following spheroid collection, spheroid were dissociated to singles cells using TryplE. Single cells were collected following centrifugation at 500 × g for 10 min and stained live with CellROX reagent at a final concentration of 1000 nM for 30 min. The stained cells were filtered through a strainer cap into a 12 × 75 FACS tube. The stained cells were analyzed on a BD FACSCanto Flow cytometer for cell cycle at the Duke Cancer Institute Flow Cytometry core facility. The data were analyzed using the FlowJo software (Ashland, OR), where the measurements were fitted into the Watson pragmatic model.

### Protease Activity Assay

DQ gelatin fluorescence in the presence of proteases. Stock solutions were made at 1 mg mL^−1^ in diH_2_O, according to manufacturer's instructions. DQ‐gelatin was co‐encapsulated within Gel‐Dex at a final concentration of 100 µg mL^−1^. Following treatment and/or laccase addition for 6 h on day 2, fluorescence intensity was quantified on day 3 in a confocal microscope using live‐cell imaging incubator. Intensity quantification was performed in NIS Elements (Nikon). An ROI was drawn manually including only the sprouts of each spheroid (and excluding its core). For the media readings, 80 µL of each hydrogel were extracted and fluorescence was measured at 487/528 nm (excitation/emission).

### Small‐Molecule Inhibition Studies

DPI stock solution at 5 mM in DMSO was used to inhibit angiogenic sprouting on day 2 during the 6‐h laccase incubation. DPI was dissolved at a final concentration of 20 µM in cell culture media supplemented with 50 ng mL^−1^ VEGF and 25 ng mL^−1^ FGF. After 6 h, the solution was replaced with fresh medium. GM6001 stock solution was prepared at 100 mM in DMSO and was used to inhibit angiogenic sprouting on day 2 following the 6‐h laccase incubation. GM6001 was dissolved at a final concentration of 1 mM in cell culture media supplemented with 50 ng mL^−1^ VEGF and 25 ng mL^−1^ FGF. After 6 h, the solution was replaced with fresh medium. DMOG stock solution was made in diH_2_O and added within cell culture medium at a final concentration of 1mM on top of each hydrogel. DMOG‐medium solution was replaced after 6 h with fresh medium.

### siRNA Transfection

ECFCs were transfected with SMARTpool:siGENOME HIF1A, EPAS1 and Non‐Targeting siRNA 1 according to the manufacturer's protocol. Briefly, cells were seeded on a six‐well plate overnight and treated with 50 nM siRNA for 24 h. The knockdown was confirmed via quantitative real‐time fluorescence polymerase chain reaction (qRT‐PCR) after 24 h (see Figure S5, Supporting Information) and transfected cells were used to prepare endothelial spheroids.

### RNA Extraction and qRT‐PCR

Total RNA was extracted using TRIzol reagent and purified using the QIAGEN RNaeasy Mini Kit. RNA quality was assessed using a nanodrop spectrophotometer. Complementary DNA (cDNA) was generated using the GoScript Reverse Transcriptase Kit (Promega). The TaqMan Universal PCR Master Mix and Gene Expression Assays were used for genes of interest. TaqMan PCR was performed using the QuantStudio 5 PCR System. The ΔΔCT method was used to calculate the amplification difference between samples as normalized to the endogenous control gene GAPDH.

### Western Blotting

ECFCs were lysed using RIPA Buffer (Thermo Fisher) with 1X Protease and Phosphatase Inhibitor Cocktail (Thermo Fisher Scientific). Protein was quantified using the BCA Assay (Thermo Fisher Scientific). 20 µg of protein from each sample was boiled at 95 °C for 5 min, then loaded into a 4–12% Bis‐Tris Protein Gel (Bio‐Rad). Proteins were transferred to a PVDF membrane (BioRad, Hercules, CA) via wet transfer overnight at 4 °C. Protein transfer was confirmed using Ponceau‐S stain. Membranes were blocked in 5% milk for 1 h at room temperature and then incubated in primary antibody overnight (1:1000) with gentle agitation at 4 °C. Membranes were washed three times for 10 min in Tris‐buffered saline with 0.1% Tween‐20 (TBST), then incubated for 1 h at room temperature with anti‐rabbit IgG, HRP‐linked antibody (Cell Signaling Technologies Danvers, MA) with gentle agitation. The membrane was washed three times with TBST to visualize the protein of interest substrate (Thermo Fisher Scientific) was added, and membranes were imaged using chemiluminescent blot imager (Biorad). Blots were analyzed using ImageJ (public domain). Following imaging of the protein of interest, blots were stripped in stripping buffer (containing glycine, SDS, and Tween‐20) by washing three times with buffer, twice with PBS, twice with TBST, blocked in 5% milk, then substrate was added to ensure complete removal of antibody. Bands were normalized to GAPDH levels as endogenous control.

### Statistical Analysis

The authors performed statistical analysis using GraphPad Prism 10 (GraphPad Software Inc.). This software was also used to perform *t*‐tests and one‐way ANOVA to determine significance. Replicates were indicated throughout the figure captions. Significance levels were set at *p < 0.05, **p < 0.01, ***p < 0.001, and ****p < 0.0001.

## Conflict of Interest

The authors declare no conflict of interest.

## Supporting information



Supporting Information

## Data Availability

The data that support the findings of this study are available from the corresponding author upon reasonable request.
