# Supplementary material for: Acute Three‐Dimensional Hypoxia Regulates Angiogenesis
Source: Adv Healthc Mater. 2024 Dec 2;14(2):2403860. doi: 10.1002/adhm.202403860 (PMC11729260; doi:10.1002/adhm.202403860)
Supplement: Supplementary file 1 — Supporting Information [file ADHM-14-0-s001.pdf]

ADVANCED HEALTHCARE MATERIALS

Supporting Information

for *Adv. Healthcare Mater.*, DOI 10.1002/adhm.202403860

Acute Three-Dimensional Hypoxia Regulates Angiogenesis

*Dimitris Ntekoumes, Jiyeon Song, Haohao Liu, Connor Amelung, Ya Guan and Sharon Gerecht**

Acute three-dimensional hypoxia regulates angiogenesis

Authors: Dimitris Ntekoumes[1, 2], Jiyeon Song[1], Haohao Liu[1], Connor Amelung[1], Ya Guan[1], Sharon Gerecht[1, *]

Affiliations:
1. Department of Biomedical Engineering, Duke University, Durham, NC, 27708, USA
2. Department of Chemical and Biomolecular Engineering, Johns Hopkins University, Baltimore, MD 21218, USA.
* Author to whom correspondence should be addressed

Supporting information

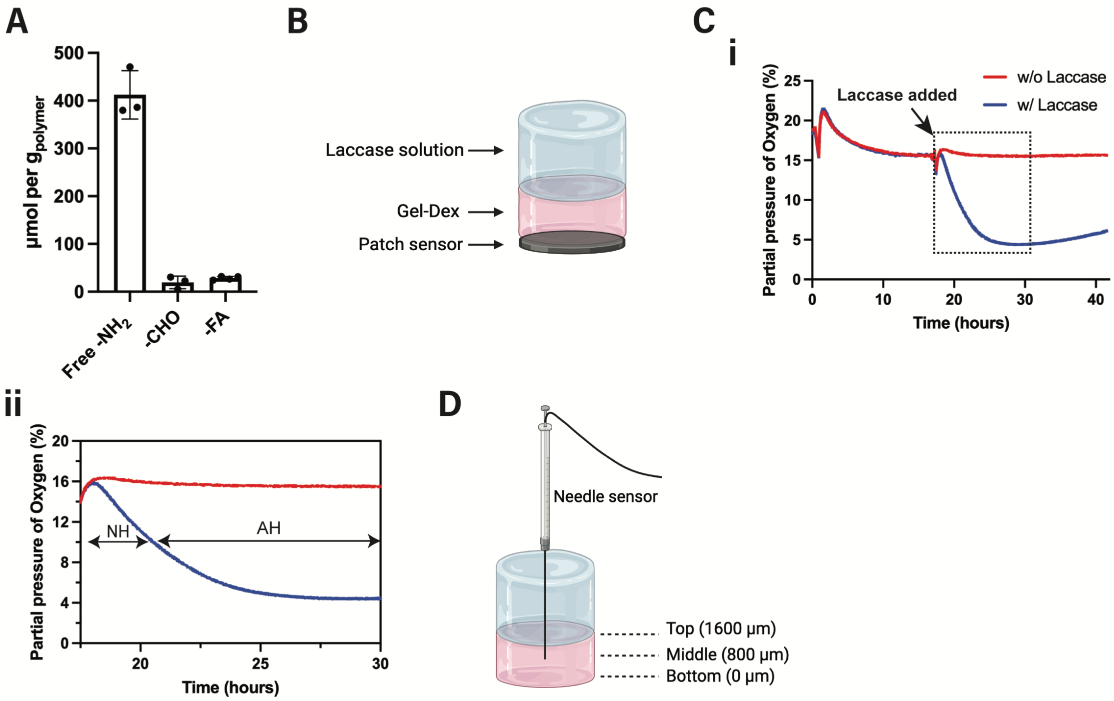

Figure S1. A. Quantification of the functional groups of Gel-Dex (Gel-FA; free -NH2, -FA and Dex-CHO; -CHO). **B.** Experimental setup for measuring partial pressure of O_2 noninvasively. **C. i-** Noninvasive dissolved O_2 readings at the bottom of the hydrogel without removing the laccase solution after 6 hours incubation ii- O2 readings within the highlighted dotted region of i-. For approximately 3 hour of laccase incubation, Gel-Dex remains nonhypoxic (NH)**.** Acute hypoxia (AH) can be sustained for over 10 hours without removing laccase. **D.** Experimental setup for invasive measurements of partial pressure of O_2 throughout the hydrogel.

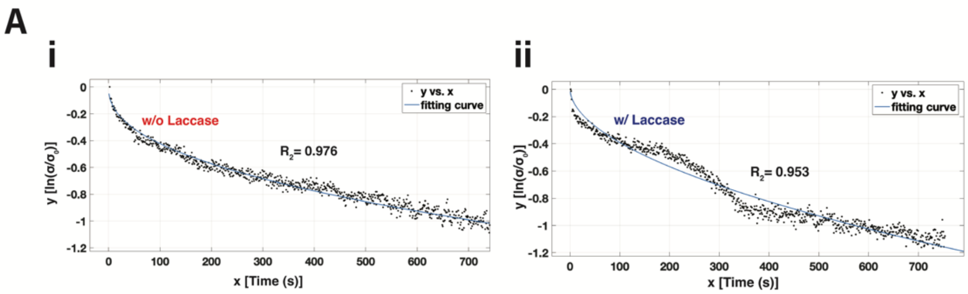

Figure S2. A. Stress relaxation behaviors for Gel-Dex without **(-i)** and with **(-ii)** could be closely fitted to that of an empirical stretched exponential function, which agrees with the experimental data (black points).

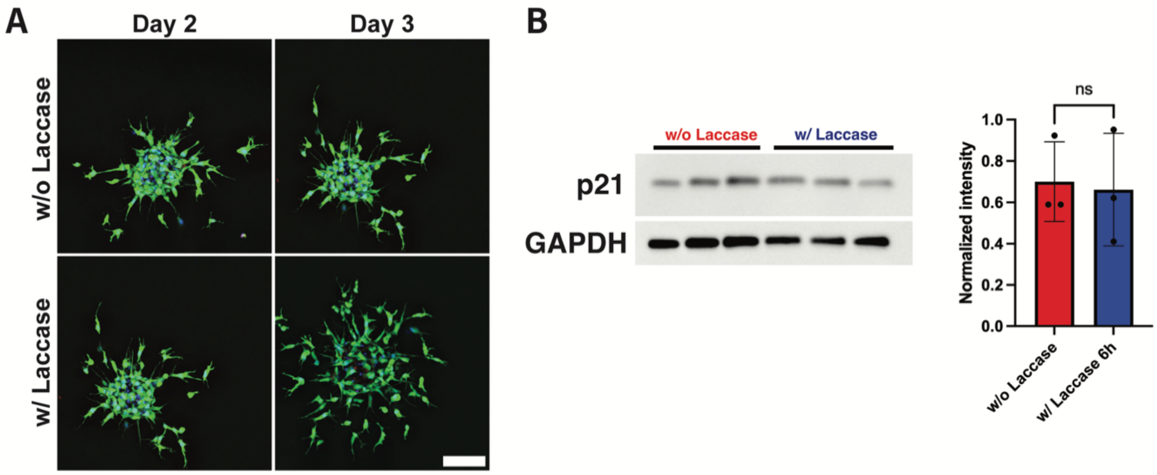

Figure S3. A. Representative LIVE (green) /DEAD (red) images of ECFC spheroids with and without laccase on day 2 and day 3. **B.** Western blot quantification of p21 expression levels on 2D ECFCs following 6-hour incubation with 2.5 U ml^{-1} laccase. ns = not significant. Scale bar is 100 μm.

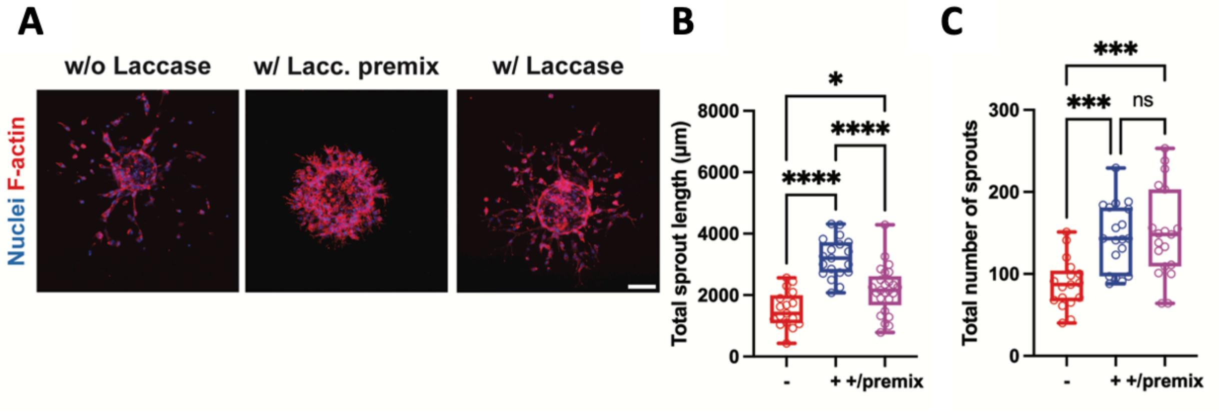

Figure S4. A) Introduction of laccase in Gel-Dex using the current (w/ Laccase) vs. the pre-gelation solution (w/ Lacc. premix) approach. Day 3 representative images of maximum intensity projection of confocal z-stack of endothelial spheroids. Quantification of the **B)** total length and **C)** number of sprouts in Gel-Dex when hypoxia in induced "on-demand" (+) and upon hydrogel formation (+/premix) compared to their nonhypoxic (-) counterparts. Scale bar is 100 μm. Significance levels were set as: *p<0.05, ***p<0.001, ****p<0.0001.

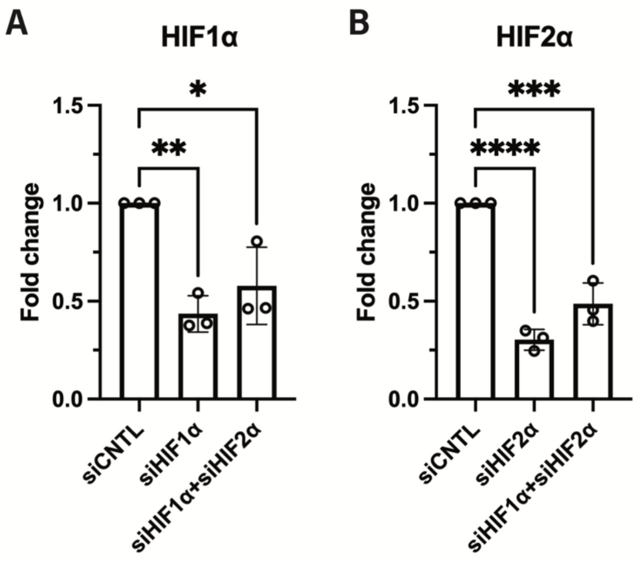

Figure S5. A, B) HIF siRNA study. RT-PCR analysis of siRNA-transfected ECFCs shows significant suppression of HIF1α and/or HIF2α compared to control siRNA. Values shown are means and ± standard deviation. Significance levels were set at: *P<0.05, **P<0.01, ***P<0.001, ****P<0.0001.

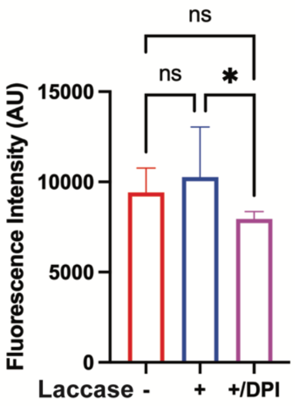

Figure S6. Fluorescence intensity readings of DQ-gelatin in the cell medium of day 3 spheroids in Gel-Dex without (-), with (+) and with the addition of laccase (+/DPI) on day 2 for 6h. (N=3 biological replicates, n=2-3 technical replicates, per condition). Significance levels were set at: *P<0.05.
